# Use of the JADAS criteria to assess efficacy of canakinumab in patients with SJIA – an analysis of 12-week pooled data

**DOI:** 10.1186/1546-0096-12-S1-P63

**Published:** 2014-09-17

**Authors:** A Ravelli, HI Brunner, N Ruperto, P Quartier, A Consolaro, N Wulffraat, K Lheritier, C Gaillez, A Martini, DJ Lovell

**Affiliations:** 1 PRINTO-Istituto Gaslini, Genova, Italy; 2 PRCSG, Cincinnati, Ohio, USA; 3 Necker-Enfant Malades Hospital, Paris, France; 4 UMC Utrecht, Utrecht, Netherlands; 5 Novartis Pharma AG, Basel, Switzerland

## Introduction

The composite score JADAS^1, 2^ 27-CRP (J27), 10-CRP (J10), and cut-off values for inactive (ID), low (LDA), moderate (MDA) and high disease activity (HDA) were designed to monitor the level of disease activity in all JIA subtypes^1^. The efficacy of canakinumab (CAN), a selective, human, anti-IL-1β monoclonal antibody, was previously demonstrated in SJIA in phase III trials using aACR-JIA response criteria^3^.

## Objectives

To assess the level of disease activity in CAN-treated SJIA patients, using J10 and J27 in a 12-week pooled (phase III studies) data set.

## Methods

Patients, 2–19 years of age, with active SJIA were enrolled and received sc CAN 4 mg/kg. This post-hoc analysis focuses on a 12-week pooled dataset (from 3 phase III studies) in a total of 178 CAN-naïve patients, assessing the J10 and J27 scores at Days (D) 15, 29, 57, 85, and applies the appropriate cut-off values for ID, LDA, MDA and HDA.

## Results

At baseline, the median [Q1,Q3] J10 for completer patients (i.e. patients who complete 12 weeks treatment) was 29.1 [23.1,33.2], and the median change [Q1,Q3] from baseline at D15 and D85 was -19.4 [-25.7,-13.4] and -21.2 [-27.7,-16.7], respectively. Results for J27 were very similar. The disease status at all time points for J10 and J27 are reported in Table 1. Median change from baseline at each time point was consistent between the completers and the full analysis dataset for J10 and J27.

**Table 1:** J10 and J27-related disease criteria on all patients

%	Disease State*	Baseline N=178	D15 N=172	D29 N=157	D57 N=131	D85 N=125
**J10**	ID	0.0	18.0	26.8	34.4	33.6

	LDA	0.0	14.0	11.5	16.8	24.8

	MDA	0.6	19.2	19.7	20.6	16.8

	HDA	99.4	48.8	42.0	28.2	24.8

**J27**	ID	0.0	18.0	26.8	34.4	33.6

	LDA	0.0	14.0	11.5	16.8	25.6

	MDA	0.6	15.1	16.6	17.6	12.8

	HDA	99.4	52.9	45.2	31.3	28.0

*Cut-off values for ID, LDA, MDA and HDA, respectively for J10: ≤1, >1-≤3.8, >3.8 - ≤10.5, >10.5; and for J27: ≤1, >1 - ≤3.8, >3.8 - ≤8.5, >8.5

## Conclusion

In the pooled 12-week dataset, there was a dramatic reduction in disease activity from baseline to D85, with much of the reduction taking place by D15 onwards in both completers and in the full analysis set. An increasing proportion of CAN patients achieved ID or LDA - according to J10 and J27 - in the first 12 weeks of treatment, despite corticosteroid tapering, a finding consistent with that using the previous ID definition from the phase III trials. These data confirm the early onset of effect as well as the short-term and sustained efficacy over 12 weeks of canakinumab, and suggest that JADAS may represent a useful tool to monitor treatment response.

## Disclosure of interest

A. Ravelli Grant / Research Support from: Pfizer, Consultant for: Abbvie, Bristol Myers Squibb, Novartis, Pfizer, Roche and Johnson & Johnson, Speaker Bureau of: Abbvie, Bristol Myers Squibb, Novartis, Pfizer, Roche and Johnson & Johnson, H. Brunner Consultant for: Novartis, Genentech, Pfizer, UCB, AstraZeneca, Biogen, Boehringer-Ingelheim, Regeneron, Paid Instructor for: Novartis, Speaker Bureau of: Novartis, Genentech, N. Ruperto Grant / Research Support from: To Gaslini Hospital: Abbott, Astrazeneca, BMS, Centocor Research & Development, Eli Lilly and Company, "Francesco Angelini", Glaxo Smith & Kline, Italfarmaco, Novartis, Pfizer Inc., Roche, Sanofi Aventis, Schwarz Biosciences GmbH, Xoma, Wyeth Pharmaceuticals Inc.,, Speaker Bureau of: Astrazeneca, Bristol Myers and Squibb, Janssen Biologics B.V.,Roche, Wyeth/Pfizer, P. Quartier Grant / Research Support from: Abbvie, BMS, Chugai-Roche, Novartis, Pfizer and SOBI, Consultant for: Abbvie, Chugai-Roche, Novartis, Pfizer, Servier and SOBI,, Speaker Bureau of: Chugai-Roche, MEDIMMUNE, Novartis, Pfizer, A. Consolaro Consultant for: Novartis, N. Wulffraat Grant / Research Support from: Abbvie, Roche, Consultant for: Novartis, Pfizer, Roche, K. Lheritier Shareholder of: Novartis, Employee of: Novartis, C. Gaillez Shareholder of: Novartis, Employee of: Novartis, A. Martini Grant / Research Support from: The Gaslini Hospital, which is the public Hospital where I work as full time employee, has received contributions to support the PRINTO research activities from the following companies: Bristol Myers and Squibb, Centocor Research & Development, Glaxo Smith & Kline,Novartis,Pfizer Inc, Roche, Sanofi Aventis, Schwarz Biosciences GmbH, Speaker Bureau of: Abbott, Bristol MyersSquibb, Astellas, Behringer, Italfarmaco, MedImmune, Novartis, NovoNordisk, Pfizer,Sanofi,Roche, Servier, D. Lovell Grant / Research Support from: National Institutes of Health- NIAMS, Consultant for: Astra-Zeneca, Centocor, Amgen, Bristol Meyers Squibb, Abbott, Pfizer, Regeneron, Roche, Novartis, UBC, Forest Research Institute, Horizon, Johnson & Johnson, Speaker Bureau of: Novartis, Roche.
